# Supplementary material for: Factors Influencing Patient Satisfaction and Loyalty as Perceived by Dentists and Their Patients
Source: Dent J (Basel). 2023 Aug 28;11(9):203. doi: 10.3390/dj11090203 (PMC10529870; doi:10.3390/dj11090203)
Supplement: Supplementary file 1 [file dentistry-11-00203-s001.zip › Supplementary File S1_Patient questionnaire.pdf]

Factors of patient satisfaction in dental care

The purpose of this research questionnaire is to gather information on patient-dentist relationship and explore what influences patients' satisfaction with their dentist and dental visits/treatments. Although we would be grateful if you could fully complete the questionnaire, you can skip any question you do not wish to answer. The questionnaire is anonymous. Please do not provide any information that could identify you. However, you may leave comments on the backside if you feel that you would like to share something with us. Thank you for your help and cooperation.

Answer the following questions by circling the **number that best reflects your feelings**.
Options: 1= Strongly disagree, 2= Disagree, 3= Neither agree nor disagree, 4= Agree, 5= Completely agree.

1. **Sex:**
   - ☐ Female
   - ☐ Male

2. **Age:** ………… (years)

3. **Highest level of education:**
   - ☐ Primary school
   - ☐ Secondary school
   - ☐ University/High school
   - ☐ Postgraduate (PhD)

4. **Type of residence:**
   - ☐ Capital
   - ☐ County seat
   - ☐ Other city/town
   - ☐ Village
   - ☐ Farm, hamlet

5. **Name the dental office you regularly attend:**

   ……

6. **The people where I currently get my dental service matter to me (1).**

Strongly disagree 1 2 3 4 5 Completely agree

7. **I have developed a personal relationship with my current dentist (2).**

Strongly disagree 1 2 3 4 5 Completely agree

8. **I trust this dentist's judgments about my medical care (2).**

Strongly disagree 1 2 3 4 5 Completely agree

9. I'm confident that my dentist knows me (3).
Strongly disagree 1 2 3 4 5 Completely agree

10. I'm confident that my dentist knows my medical records (3).
Strongly disagree 1 2 3 4 5 Completely agree

11. I would recommend my dentist to others (2).
Strongly disagree 1 2 3 4 5 Completely agree

12. I am very committed to continuing a relationship with my dentist (4).

Strongly disagree 1 2 3 4 5 Completely agree

13. For me, the costs in time / money / effort to switch dentists are high (2).
Strongly disagree 1 2 3 4 5 Completely agree

14. I possess good knowledge of health care services (5).
Strongly disagree 1 2 3 4 5 Completely agree

15. I am quite experienced in the health care area (5).
Strongly disagree 1 2 3 4 5 Completely agree

<u>The questions below refer to your last visit at your dentist</u>

16. How many times have you been to your current dentist? < 10 times / > 10 times

17. The dentist was interested in my worries about my problem (3).
Strongly disagree 1 2 3 4 5 Completely agree

18. The dentist was interested when I spoke about my symptoms (3).
Strongly disagree 1 2 3 4 5 Completely agree

19. The dentist was interested in the effects of the problem on my family or private life (3).
Strongly disagree 1 2 3 4 5 Completely agree

20. The dentist explained clearly what the problem was (3).
Strongly disagree 1 2 3 4 5 Completely agree

21. The dentist was careful to explain the treatment plan (3).
Strongly disagree 1 2 3 4 5 Completely agree

22. The dentist encouraged me to ask questions about my treatment (6).
Strongly disagree 1 2 3 4 5 Completely agree

23. The dentist used words that were understandable in talking about my dental care (6).
Strongly disagree 1 2 3 4 5 Completely agree

24. The dentist told me what s/he was going to do before starting the procedure (6).
Strongly disagree 1 2 3 4 5 Completely agree

25. I am satisfied with the duration of the appointments (6).
Strongly disagree 1 2 3 4 5 Completely agree

26. I am satisfied with the frequency of the appointments (6).
Strongly disagree 1 2 3 4 5 Completely agree

27. I am satisfied with the quality of the treatment (6).
Strongly disagree 1 2 3 4 5 Completely agree

28. I am satisfied with the explanation given by the dentist (6).
Strongly disagree 1 2 3 4 5 Completely agree

29. The dentist offered me the choice between more than one treatment plan (3).
Strongly disagree 1 2 3 4 5 Completely agree

30. The dentist discussed the treatment plan with me (3).
Strongly disagree 1 2 3 4 5 Completely agree

31. The dentist reached agreement with me on the treatment plan (3).
Strongly disagree 1 2 3 4 5 Completely agree

The sources of the items (see in brackets after the items)

1. Burnham T, Frels J, Mahajan V. Consumer switching costs: A typology, antecedents, and consequences. *J Acad Mark Sci*. 2003;31:109-26.

2. Platonova EA, Kennedy KN, Shewchuk RM. Understanding patient satisfaction, trust, and loyalty to primary care physicians. *Med Care Res Rev*. 2008;65(6):696-712.

3. Little P, Everitt H, Williamson I, Warner G, Moore M, Gould C, et al. Observational study of effect of patient centredness and positive approach on outcomes of general practice consultations. *BMJ*. 2001;323(7318):908-11.

4. Wang HL, Huang JY, Howng SL. The effect on patient loyalty of service quality, patient visit experience and perceived switching costs: lessons from one Taiwan university hospital. *Health Serv Manage Res*. 2011;24(1):29-36.

5. Sharma N, Patterson P. Switching costs, alternative attractiveness and experience as moderators of relationship commitment in professional, consumer services. *Int J Serv Ind Management*. 2000;11(5):470-90.

6. Al-Mobeeriek A. Dentist-patient communication as perceived by patients in Riyadh, Saudi Arabia. *Int J Occup Med Environ Health*. 2012;25(1):89-96.
