# Supplementary material for: Factors Influencing Patient Satisfaction and Loyalty as Perceived by Dentists and Their Patients
Source: Dent J (Basel). 2023 Aug 28;11(9):203. doi: 10.3390/dj11090203 (PMC10529870; doi:10.3390/dj11090203)
Supplement: Supplementary file 1 [file dentistry-11-00203-s001.zip › Supplementary File S2_Dentist questionnaire.pdf]

Factors of patient satisfaction in dental care

The purpose of this research questionnaire is to gather information on patient-dentist relationship and identify those factors that, in your opinion, influence your patients' satisfaction with their dental treatments. Although we would be grateful if you could fully complete the questionnaire, you can skip any question you do not wish to answer. The questionnaire is anonymous. Please do not provide any information that could identify you. However, you may leave comments on the backside if you feel that you would like to share something with us. Thank you for your help and cooperation.

Answer the following questions by circling the **number that best reflects your feelings**.
Options: 1= Strongly disagree, 2= Disagree, 3= Neither agree or disagree, 4= Agree, 5= Completely agree.

1. **Sex:**
   - ☐ Female
   - ☐ Male

2. **Age:** ………… (years)

3. **For how many years have you been working as a dentist?** ………… (years)

4. **Where is the dental office you work for located?**
   - ☐ Capital
   - ☐ County seat
   - ☐ Other city/town
   - ☐ Village
   - ☐ Farm, hamlet

5. **I think patients trust their dentist's judgments about their medical care.**

Strongly disagree 1 2 3 4 5 Completely agree

6. **I think my patients would recommend me to others.**

Strongly disagree 1 2 3 4 5 Completely agree

7. **I think my patients are very committed to continuing a relationship with me.**

Strongly disagree 1 2 3 4 5 Completely agree

8. **Most of the patients possess good knowledge of health care services.**

Strongly disagree 1 2 3 4 5 Completely agree

9. **The most important characteristic(s) influencing satisfaction with the dentist's care for patients is/are:**

……

……

10. It matters to the patients that their dentist shows interest when they speak about their symptoms.

Strongly disagree 1 2 3 4 5 Completely agree

11. It matters to the patients that their dentist explains clearly what the problem is.

Strongly disagree 1 2 3 4 5 Completely agree

12. It matters to the patients that the dentist is careful to explain the treatment plan.

Strongly disagree 1 2 3 4 5 Completely agree

13. It matters to the patients that the dentist encourages them to ask questions about their treatment.

Strongly disagree 1 2 3 4 5 Completely agree

14. It matters to the patients that their dentist uses words that are understandable in talking about their dental care.

Strongly disagree 1 2 3 4 5 Completely agree

15. It matters to the patients that their dentist tells them what s/he is going to do before starting the procedure.

Strongly disagree 1 2 3 4 5 Completely agree

16. The duration of an appointment matters to the patients.

Strongly disagree 1 2 3 4 5 Completely agree

17. The frequency of appointments matters to the patients.

Strongly disagree 1 2 3 4 5 Completely agree

18. The quality of the treatment matters to the patients.

Strongly disagree 1 2 3 4 5 Completely agree

19. I think it is important to offer my patients the choice between more than one treatment plan.

Strongly disagree 1 2 3 4 5 Completely agree

20. It matters to the patients that their dentist discusses the treatment plan with them.

Strongly disagree 1 2 3 4 5 Completely agree

21. It matters to the patients that their dentist reaches agreement with them on the treatment plan.

Strongly disagree 1 2 3 4 5 Completely agree
